# Feminist perspectives on environmental justice and health in Jamaica

**DOI:** 10.3389/fsoc.2024.1347649

**Published:** 2024-06-07

**Authors:** Neena Albarus, J’Anna-Mare Lue, Erin Kerrison, Maya Carrasquillo

**Affiliations:** ^1^School of Social Welfare, University of California, Berkeley, Berkeley, CA, United States; ^2^Liberatory Infrastructures Laboratory, Department of Civil and Environmental Engineering, University of California, Berkeley, Berkeley, CA, United States

**Keywords:** environmental racism, Jamaica, Caribbbean feminism, environmental justice, intersectionality

## Abstract

Jamaica is an island nation with a history that is informed by Taino settlement, European colonisation, chattel slavery, disinvestment, and continued extractivism. This perspective paper leverages a historical analysis to explore environmental injustices affecting the health and quality of life of Jamaicans living in Jamaica. This article hopes to contribute to a growing but limited body of scholarly research that contends with environmental and climate justice in the context of the Caribbean. In discussing a lack of critical environmental infrastructure, such as reliable solid waste management, and the impacts of extractive industries, such as bauxite mining, the paper intends to highlight the environmental, public health, and social harms that are produced. Employing an intersectional approach grounded in Black feminist epistemology put forward by Patricia Hill Collins, the authors use their lived experiences as a source of knowledge. The paper analyses how these environmental injustices harm Jamaican communities at large but underscores the compounded challenges faced by Jamaican women who experience marginalisation on the basis of gender, urban/rural residency, and class. The paper concludes by urging researchers, policymakers, regulatory bodies, and other stakeholders to conduct further research and create sustainable and equitable environmental standards that have considerations for environmental injustice in Jamaica.

## 1 Introduction

Environmental justice is marked by patterns of inequality, power imbalances, and systemic disadvantages (Newell, 2005). As such, this article positions itself within the context of a global predicament, contributing a vital perspective to the ongoing discourse on environmental justice. This paper seeks to build on the small but growing body of work around environmental justice in the Caribbean produced by thinkers such as Esther Figueroa, Robert Connell, Anne-Teresa Birthwright, April Karen Baptiste, Stacy-ann Robinson, R. Anna Hayward, Debra D. Joseph, and Rachael Baptiste-Garrin (Hayward and Joseph, 2018; Figueroa, 2019; Connell, 2020; Birthwright, 2022; Baptiste and Baptiste-Garrin, 2023). The aim of this work is to contextualise the impact of environmental injustice on women’s health in Jamaica offering a Caribbean perspective that considers unique socio-economic and historical factors. Additionally, through this localised lens, the paper aims to inform targeted strategies for equitable environmental standards in the region. The paper will discuss the unique challenges faced by Jamaican women concerning environmental health, including exposure to pollutants, access to resources, and the broader implications for their well-being. The paper will also highlight nuances of the socioeconomic and historical factors shaping these challenges, recognizing the intersectionality of race, class, and gender within the environmental justice paradigm. The paper is motivated by the urgency of addressing this specific facet of environmental justice, emphasising the adverse repercussions on Jamaican women’s health and Jamaica’s ecological health.

Environmental justice attends to environmental burdens and lack of access to environmental resources by experienced marginalised communities typically along axes of difference of class, race, gender, and geography (Ali and Kamraju, 2023). A critical piece in the struggle for environmental justice is acknowledging environmental racism, which was first defined by Robert D. Bullard as “any policy, practice or directive that differentially affects or disadvantages (where intended or unintended) individuals, groups or communities based on race” (Bullard, 1993). Bullard, who is known as the “father of environmental justice,” attributes ecological inequalities to land use planning, distribution of wealth as well as predatory real estate and housing practices. Anti-blackness, which both dehumanises and systematically marginalises Black people, is a pervasive form of racism that is a key driver of environmental injustice (Vasudevan and Smith, 2020). Environmental injustice, for this paper, highlights the intersection of race, poverty, and geography as factors that determine the extent to which individuals are exposed to pollution and environmental hazards. Studies highlight the complex factors contributing to environmental injustices in the global south, emphasising power dynamics and the need for stronger institutions (Cifuentes and Frumkin, 2007). Additionally, broader social and political structures contribute to environmental injustices in the global south, including the global political economy and North–South power differentials leading to unequal resource distribution, economic disparities, and limited decision-making access (Cifuentes and Frumkin, 2007). Informing this work is the proposition of Baptiste and Robinson that Caribbean environmental justice theory is undergirded by the themes of “coloniality, sovereignty, and resistance” (Baptiste and Robinson, 2023).

Jamaica is a small island in the Caribbean originally inhabited by the Taino people. However, Jamaica the small island developing state (SIDS) was a result of centuries of colonial conquest which decimated Indigenous populations, trafficked Africans and enslaved their labour to commit ecocide to develop a plantation economy based on the production of sugar. This is the foundation of environmental injustice in Jamaica which is inherently an issue of racial justice. Moreover, the legacies of colonization, enslavement, and genocide have profoundly shaped the environmental trajectory, with race exerting a lasting influence on individuals’ lives (Canham, 2023). Slavery in the Caribbean had enduring effects on the environment, primarily due to practices employed to bolster the plantation economy’s mono-crop culture. For instance, large-scale sugar plantations were derived from extensive deforestation and destruction of indigenous plant and animal species (Ferdin and Anthony, 2022). Furthermore, changes in land use and water management practices adversely affected local ecosystems as plantations required significant amounts of water (Hauser, 2017). The socio-economic inequities that originated with slavery also contribute to unsustainable environmental practices due to poverty and lack of resources as well as a severing of relationships with land.

During the era of colonialism, other natural resources such as timber, bauxite, and minerals were also exploited by European powers for their economic benefit (Knight and Beckford, 1973). This exploitation resulted in significant environmental degradation, including deforestation, soil erosion, and water pollution (Jaffe, 2006). Even after Jamaica gained independence in 1962, the country continued to face environmental challenges, exacerbated by rapid urbanisation and industrialisation. As a result, many low-income communities residing in these places were established in close proximity to industrial plants, waste disposal sites, and other sources of pollution. These patterns of development were driven by policies and practices that prioritised the interests of politicians, wealthy individuals, and multinational corporations over the well-being of local communities such as the IMF prompted tax concessions that incentivize extractive industries coming to Jamaica without real benefits to Jamaicans with the exception of low-paying wage work (Knight and Beckford, 1973; Ali and Kamraju, 2023). One example is the preferential treatment given to multinational corporations within the Bauxite-Alumina Industry, which often received lenient environmental regulations, manipulated accounting prices, and underpaid employee wages (Velasquez, 1995).

The historical legacy of colonialism and slavery in Jamaica established power structures that continue to influence environmental injustices. The initial distribution of power, where colonial powers exploited resources for economic gain, laid the foundation for the unequal environmental burdens borne by Black communities. The exploitation of natural resources in developing nations, driven by global economic interests, mirrors the power imbalances seen at the intersection of race, gender, and class. Powerful global actors, often from wealthier nations, exploit resources in less affluent regions, contributing to environmental degradation and disproportionately affecting marginalised communities.

While we are concerned with the health of Jamaicans more broadly, the health of women is important given the social position of women in Jamaica where family structure is seen as matrifocal although society is largely patriarchal (Massiah, 1983; O’Connor, 2014; Renaud, 2020). At the household level and at the societal scale we see women experiencing triple burden (Griffin, 2017) and triple oppression (Jones, 1949) where the brunt of household labour and family/social life often falls on the shoulders of Jamaican women in addition to the expectation of informal and wage work in addition to discrimination based on race (and colour), class, and gender. It is within this context that we theorise outside of the home and into the realm of social and geopolitical arrangements where we arrive at the understanding that “the political ecologies and political economies of survival for people living in extractive zones are embodied matters. Neoliberal expropriations of land and other wealth inscribes itself on women’s bodies, even as they seek means to maintain lives of dignity. Extractive logics ensnare women’s bodies in particular racialized and gendered fashion” (Murrey and Mollett, 2023). In addition to engagement with literature, both the first and second authors are Jamaican women who have lived in Jamaica for over 15 years, and are currently pursuing graduate education in the U.S. This work is also grounded in Black feminist epistemology, namely “lived experience as criterion for meaning” (Collins, 2000).

Therefore, an intersectional approach is crucial to understanding and addressing environmental injustice. Intersectionality recognizes that different social identities, such as race, class, gender, and others, interact and compound the experiences of oppression and privilege that individuals face (Rice et al., 2019). Originating from feminist discourse on the experiences of Black women in the United States, intersectionality highlights the limitations of addressing oppression through a single identity construct that overlooks the nuanced intragroup variations within diverse marginalised contexts (Crenshaw, 1991). By considering these intersecting identities, we can better comprehend the unique experiences of marginalised communities and develop comprehensive strategies to combat systemic injustices (Joseph, 2015). In expanding this framework to the domain of environmental justice, it is imperative to scrutinise its ramifications on the health of low-income Jamaican women.

In the context of Jamaica, the intersections of race, gender, and class shape women’s experiences and the extent to which they are affected by environmental hazards. In terms of race, the country’s history of colonialism and slavery has led to the marginalisation of Black communities, who are disproportionately affected by environmental hazards. This has been exacerbated by the legacy of racial discrimination and inequality, which has resulted in uneven distribution of resources and access to essential services such as clean water, health care, and education for Black women. Limited representation and participation in decision-making processes contribute to the perpetuation of environmental injustices, as their perspectives and needs may be overlooked by wider society.

## 2 Solid waste management

Effective solid waste management is an area of critical concern for maintaining public health and ecological health. Improper waste management leads to the pollution of environmental media—air, soil, and water leading to negative human health outcomes. Studies have shown associations between residential proximity to waste facilities with adverse pregnancy outcomes and respiratory diseases (Brender et al., 2011). Additionally, poorly managed waste disposal sites can contaminate water resources, reducing the availability of safe drinking water (Abubakar et al., 2022). Soil contamination from ineffective waste management can pose health risks through exposure to soil as well as through foodways (Yeilagi et al., 2021; Siddiqua et al., 2022).

Jamaica, like much of the Caribbean, lacks adequate waste management infrastructure which has been attributed to deficits in capital, public policy, and technical resources (Phillips and Thorne, 2013). Solid waste is managed by the National Solid Waste Management Authority (NSWMA) which currently operates eight active disposal sites—Retirement, Myersville, Martins Hill, Tobalski, Haddon, Church Corner, Doctor’s Wood, and Riverton (National Solid Waste Management Authority, 2023). The waste disposal sites are management dump sites not considered engineered landfills and are unlined and primarily uncovered (Linton, 2022). In 2021, the NSWMA collected over 990,000 tonnes of solid waste (Linton, 2022; Planning Institute of Jamaica, 2022).

In 2018, 70% of Jamaican households utilised formal disposal methods for their solid waste management which is defined as “formal disposal methods include a regular/irregular public/private collection system and dumping at a municipal disposal site.” Over 27% of all Jamaican households managed their waste by burning. Approximately 50% of rural households burned their trash. Illegal dumping and burying solid waste are also other disposal methods that are less practised (Planning Institute of Jamaica and Statistical Institution of Jamaica, 2019). Access to solid waste services is more readily available in urban areas and higher-income communities. Burning waste is a more common method of waste disposal in rural regions and low-income communities (Planning Institute of Jamaica, 2007; Post and Mihelcic, 2010; The Auditor General’s Department Jamaica, 2022). In communities where solid waste management is inadequate, resources such as safe drinking water and clean air become scarce as the practice of dumping and burning pollute both waterways and air. Jamaican women, responsible for household well-being, may struggle to access essential resources for themselves and their families. Rural and/or poor Jamaican women who are typically responsible for household waste disposal experience a unique vulnerability to exposure to pollutants while burning waste due to the higher rates of burning in rural and low-income communities attributed to a lack of sanitation services (Jamaica Gleaner, 2015; Planning Institute of Jamaica and Statistical Institution of Jamaica, 2019). This phenomenon exacerbates existing socio-economic disparities, as women in these areas contend with additional burdens in securing basic necessities hence the need for an intersectional approach to the solid waste management issues in Jamaica.

However, environmental injustice from ineffective solid waste management is not only a rural issue. The Kingston Metropolitan Area (KMA), which is heavily industrialised, serves as an illustrative example. Numerous factories, power plants, and other sources of pollution are located within the KMA, many of which are in or near low-income communities such as Riverton City, one of Jamaica’s largest informal settlements (Duncan, 2018). Residents of Riverton City and neighbouring communities have long voiced concerns about health issues linked to pollution exposure. In 2015, a fire broke out at the Riverton City landfill, emitting toxic smoke that adversely affected nearby communities, leading to respiratory problems and other health complications (National Environment & Planning Agency, 2015; Office of the Public Defender, 2016). Diana McCaulay, founder of The Jamaica Environment Trust (JET) describes area around Riverton as a sacrifice zone highlighting that even Jamaica’s ‘formal solid waste management disposal’ distributes environmental harm unevenly—while some Jamaicans benefit from waste collection services, the communities living beside the Riverton dump are left to deal with the major environmental health concerns (McCaulay, 2018). Women, often primary caregivers and homemakers, may be more exposed to the immediate health risks associated with pollution, as they navigate their daily responsibilities in close proximity to polluted environments. Furthermore, women in informal settlements near waste disposal sites often engage in informal economic activities, such as small-scale businesses or agriculture. The environmental hazards resulting from improper waste management can pose occupational risks for these women. Exposure to contaminated water, soil, and air may impact not only their health but also their livelihoods.

JET has emphasised that the Riverton dump remains one of the most substantial threats to public health for individuals living and working in its proximity, calling for an urgent health impact survey of the communities surrounding the disposal site (Baines, 2018). Furthermore, the presence of waste can attract pests and disease vectors, increasing the risk of vector-borne diseases and other health hazards. Inadequate waste management infrastructure in these communities further compounds the problem, as it contributes to the spread of diseases and the accumulation of environmental pollutants. It is important to note that the situation at the Riverton City landfill is not an isolated case, Retirement Dump in Montego Bay and Church Corner Dump in St. Thomas have reported fires in the past two years (Frater, 2021; Jamaica Gleaner, 2021).

The Jamaican government is currently planning to privatise the country’s solid waste management with the NSWMA acting as a regulating body. This decision to embark on Public-Private Partnerships to better manage solid waste is supported by various development centred organisations (Development Bank of Jamaica, 2022). Whereas privatising systems such as solid waste management is often touted as a cure-all solution to ineffective public services, privatised waste management is not without it is challenges as seen where lack of engagement with the public, paucity of legislation and comprehensive planning therefore limited accountability, and other institutional limitations can lead to poor and even worse outcomes in waste management (Ahmed and Ali, 2006; Kassim and Ali, 2006; Bolaane and Isaac, 2015; Ebekozien et al., 2022).

## 3 Bauxite

Bauxite is a reddish rock rich in aluminium content found in more than 20% of Jamaica’s surface area (Greenberg and Wilding, 2007). Bauxite mining in Jamaica is another significant example of the environmental challenges and potential environmental justice. This industry, which plays a major role in the country’s economy, has been associated with environmental degradation, deforestation, and displacement of local communities (Berglund and Johansson, 2004). Health studies conducted in other countries have suggested a connection between bauxite mining and processing and various health issues, including hypertension, asthma, sinusitis, and respiratory problems (Donoghue et al., 2014; Wesdock and Arnold, 2014; Lee et al., 2020). However, Jamaican authorities have often dismissed claims of illnesses related to bauxite mining and processing. They have rejected requests for compensation, medical treatment, or corrective measures for affected communities, citing a lack of statistical proof of a direct causal link between bauxite processing and the reported health issues (Williams, 2004).

The adverse effects of bauxite mining in Jamaica extend beyond environmental degradation and health issues to disproportionately impact Jamaican women, particularly those residing in marginalised and low-income communities. The proximity of these women to bauxite mining areas and landfills heightens their vulnerability to the negative health impacts of environmental hazards. Living in close proximity to bauxite mining sites exposes women to air and water pollution, toxic waste, and the release of hazardous gases.

In the effort to address the Jamaican Government’s nonresponse to the request for information about precautionary measures taken to prevent health and other dangers posed by bauxite mining to residents in several St. Ann communities, the Inter-American Commission on Human Rights (IACHR) issued a 20-day ultimatum to the Jamaican Government in December 2022. The request to the IACHR alleged that exposure to bauxite dust has resulted in various health problems, including ear, nose, and throat issues, upper respiratory tract infections, asthma, chronic obstructive pulmonary disease, rhinitis, sinusitis, eczema, impetigo, and exacerbation of heart failure (Jamaica Gleaner, 2022). The IACHR’s intervention highlights the pressing need for immediate action to address the risks posed by bauxite mining and safeguard the health and well-being of affected residents.

The adverse health outcomes resulting from exposure to environmental hazards are multifaceted. Public health studies concerning these injustices in similarly situated inequitable health access arrangements demonstrate that exposure to environmental pollutants such as lead, mercury, and polychlorinated biphenyls (PCBs) can have negative impacts on reproductive health, including infertility, miscarriages, and birth defects (Pizzorno, 2018; Kumar et al., 2019). Furthermore, the notion that mining is a necessary step for societal advancement often obscures the environmental degradation and social injustices that disproportionately affect marginalised communities.

## 4 Discussion and conclusion

While there is still much to learn about how environmental injustice specifically impacts women in Jamaica, the available evidence suggests that women from marginalised communities are likely at a higher risk of exposure to environmental hazards and may experience negative health impacts as a result. Gender norms play a pivotal role in shaping societal expectations and influencing the division of labour within communities. Low-income women in Jamaica often find themselves constrained by traditional gender roles, which dictate specific responsibilities within the household (Bolles, 1997). This includes responsibilities such as caregiving, household chores, and other forms of typically unpaid labour, which tend to limit women’s access to diverse economic opportunities (Mkwambisi et al., 2011; Glazebrook et al., 2020; Vercillo, 2020). This unequal distribution of domestic tasks not only limits women’s time and energy for economic pursuits but also reinforces traditional gender roles. Therefore, these women find themselves at the intersection where vulnerabilities converge, facing distinctive health challenges that differ from those experienced by other individuals. For example, at the household level, women tend to manage waste, and therefore there are disparate health risks associated with this role. At an institutional level, inequitable access to waste collection services in rural areas leads to more rural households using burning as the primary method of waste disposal. At a household level, gender roles where women are seen as managers of household waste and therefore the ones who burn the trash disproportionately expose women to more toxins and pollutants (Lakhani, 2007).

Environmental injustice in Jamaica is a pressing issue that disproportionately affects marginalised communities, subjecting them to elevated levels of pollution and environmental hazards. The historical legacies of colonialism, slavery, racial discrimination, and inequality have contributed to the marginalisation of Black communities. Within this context, women face additional vulnerabilities due to limited access to resources, political power, and the burden of household responsibilities. Furthermore, class exacerbates the effects of environmental injustice, compounding the challenges faced by marginalised communities. Prominent examples such as bauxite mining and the inadequate solid waste infrastructure, as seen in the Riverton City Dump fires and insufficient collection services, vividly illustrate the detrimental impact of environmental injustice on the health and well-being of affected communities. Urgent action is required from the Jamaican government and relevant stakeholders to address the risks associated with these industries and safeguard the health and well-being of affected residents. Holding power structures accountable for the consequences of environmental degradation and pollution is crucial. Amplifying the voices of marginalised communities in policy-making and decision-making processes is imperative, as is the enforcement of existing environmental laws and regulations. Future research should be conducted as there is a dearth of peer-reviewed literature contending with the environmental health implications of extractive industries such as mining and tourism as well as the lack of adequate infrastructure and environmental amenities. Researchers and other stakeholders should consider using an environmental justice lens to prioritise understanding the intersectional impacts of environmental hazards on mental health and overall well-being in the Caribbean context.

## Data availability statement

The original contributions presented in the study are included in the article/supplementary material, further inquiries can be directed to the corresponding author.

## Author contributions

NA: Conceptualization, Writing – original draft, Writing – review & editing. EK: Funding acquisition, Writing – review & editing. MC: Supervision, Writing – review & editing. J’A-ML: Conceptualization, Writing – original draft, Writing – review & editing.
